# Root Canal Morphology and Configuration of the Mandibular Canine: A Systematic Review

**DOI:** 10.3390/ijerph181910197

**Published:** 2021-09-28

**Authors:** Thomas Gerhard Wolf, Andrea Lisa Anderegg, Burak Yilmaz, Guglielmo Campus

**Affiliations:** 1 Department of Restorative, Preventive and Pediatric Dentistry, School of Dental Medicine, University of Bern, CH-3010 Bern, Switzerland; andrea.anderegg@zmk.unibe.ch (A.L.A.); burak.yilmaz@zmk.unibe.ch (B.Y.); guglielmo.campus@zmk.unibe.ch (G.C.); 2 Department of Periodontology and Operative Dentistry, University Medical Center, Johannes Gutenberg-University Mainz, 553131 Mainz, Germany; 3 Division of Restorative and Prosthetic Dentistry, College of Dentistry, The Ohio State University, Columbus, OH 43210, USA; 4 Department of Reconstructive Dentistry and Gerodontology, School of Dental Medicine, University of Bern, CH-3010 Bern, Switzerland; 5 Department of Surgery, Microsurgery and Medicine Sciences, School of Dentistry, University of Sassari, I-07100 Sassari, Italy; 6 Department of Pediatric, Prophylaxis Dentistry and Orthodontics, School of Dentistry, Sechenov University, 119991 Moscow, Russia

**Keywords:** internal morphology, mandibular canine, root canal configuration, number of canals, number of roots

## Abstract

The aim of this study was to systematically review the root canal morphology and configuration (RCC) of mandibular canines (MaCa). The review was registered in the PROSPERO database (ID-272297) and it was carried out following the PRISMA guidelines. Three electronic databases (MEDLINE via PubMed, Embase, Scopus) were searched. Randomized controlled trials, cross-sectional, cohort, comparative, evaluation and validation studies have been included. The anatomical quality assessment (AQUA) tool was used for a quality assessment of the anatomical studies. Of 910 studies retrieved from the systematic search, 28 studies investigating RCCs were included. Most MaCa were single-rooted (87.9–100%), while two-rooted MaCa were present up to 12.1%. The 1-1-1/1 (35.8–96.4%) was the most commonly reported RCC, followed by 2-2-1/1 (0.2–22.0%) and 1-2-1/1 (0.9–20.0%). A high frequency of 1-1-1/1 RCC in MaCa has been described. Most systematic review reports confirm that two-rooted MaCa are found considerably less frequently than single-rooted ones.

## 1. Introduction

Knowledge and understanding of the internal morphology of root canals is crucial for successful non-surgical as well as surgical endodontic therapy [1,2,3]. Although examination methods have improved significantly in recent decades, interest in the morphology of the three-dimensional root canal system and its importance has not diminished. In order to minimize or to avoid iatrogenic errors and failures in endodontic treatment, a precise knowledge of the anatomical relationships in the root canal system and the immediate recognition of possible deviations is of integral importance for the treating dentist [1,2,3].

Moreover, the realization that the morphological complexity of the root canal system can be obscured by the uniform and relatively simple radiological anatomy of the outer root surface is of great clinical benefit [4]. Various methods, such as staining and clearing [5,6,7,8,9,10], grinding [11], cross-sectional [12], microscopy [9,13,14], and radiographic analysis [15] have been used to study the morphology of the root canal system, with both *ex vivo/in vitro* and *in vivo* studies described in the literature.

Cone-beam computed tomography (CBCT) and micro-computed tomography (micro-CT) are the two most recently introduced investigation methods, and CBCT has been predominantly used for *in vivo* investigation of the morphology of the root canal system of mandibular canines (MaCa) [6,13,14,16,17,18,19,20,21,22,23,24,25,26,27,28,29,30,31]. Although micro-CT has already been used to examine various teeth as well as to describe the internal morphology of mandibular canines, it has not yet been used to identify the root canal configuration [3,32,33,34,35,36,37,38].

Micro-CT has emerged as a non-destructive, noninvasive, and reproducible examination method when in combination with 3D image rendering software and can be considered as the gold standard for dental research purposes [35,39]. Half a century ago, Vertucci [1] and Weine et al. [2] proposed two of what nowadays are the most commonly used methods to describe root canal configuration; they used decalcification, injection with dye, and clearing [1] or sectioning [2]. However, these methods cannot describe various configurations, as is possible with the method developed by Briseño-Marroquín et al. [3]. The use of micro-CT by Briseño-Marroquín et al. has the advantage that the classification system is descriptive and can be applied individually to the internal morphology of a particular root, rather than forcing a classification based on the system of internal morphology.

Therefore, the aim of the present paper is to provide a systematic review of the root canal configuration of mandibular canines, contributing to the morphological knowledge that is a prerequisite for successful endodontic treatment.

## 2. Materials and Methods

The protocol was registered in the international prospective register of systematic reviews (PROSPERO) system of the National Institute of Health Research of the Centre for Reviews and Dissemination of the University of York (United Kingdom) (ID-272297, 7 August 2021). The systematic review followed the preferred reporting items for systematic reviews and meta-analyses (PRISMA) guidelines [40].

### 2.1. Eligibility Criteria

Cross-sectional studies, comparative studies, evaluation and validation studies, and randomized controlled trials (RCTs) were included in the review procedure. Case reports and reviews were excluded. Furthermore, only papers containing data on root canal configuration were included in the systematic review. Exclusion criteria, therefore, included studies investigating other morphological issues than root canal configuration. All duplicates were removed; the remaining articles were examined by title and abstract, and papers were discarded after consulting the title and abstract and finding that they did not refer to the topic. The papers were then reviewed in full text; several papers were excluded after consulting the full text.

### 2.2. Information Sources and Search Strategy

Several literature searches through three electronic databases (MEDLINE via PubMed, Embase, and Scopus) were performed up to August 2021, using an ad hoc prepared string with Medical Subject Heading (MeSH) terms and keywords: (oot canal configuration OR root canal system OR root canal morphology) AND (morphology OR anatomy) AND (mandibular canine) without any restrictions. A cross-reference search in the reference list of full-text articles was performed. Grey literature has also been retrieved (http://www.opengrey.eu). (accessed on 26 August 2021).

### 2.3. Study Selection

Only publications in English were considered; duplicates and those articles deemed ineligible were excluded. Three authors (T.G.W., A.L.A. and G.C.) independently examined all abstracts of the screened papers. All articles that met the inclusion criteria were reviewed by two independent observers (T.G.W. and A.L.A.) in full text.

### 2.4. Data Collection, Summary Measures and Synthesis of Results

Information of the reports on publication date, authors, population investigated, number of specimens/patients, methodology, data on root canal configurations and number of roots were summarized.

### 2.5. Assessment of Bias across Studies

The risk of bias of the included studies was assessed with the anatomical quality assessment (AQUA) tool for the quality assessment of anatomical studies [41]. Two authors (T.G.W., A.L.A.) independently screened the articles and assessed the risk of bias using the five AQUA tool domains. In case of disagreement in the assessment, a third author (G.C.) was consulted to reach to a consensus. Each report has been judged as “low”, “high” or “unclear” in the categories: target and subject attributed, design of the study, methodology description, descriptive anatomy and reporting of outcomes. The tool contains five domains, each with a set of signaling dichotomous questions (Yes or No) to help assess and judge the risk of bias pertaining to it. If all questions of a category are “Yes”, then the risk of bias can be judged as “low”.

The list of excluded papers (Table S1), the quality assessment of the studies (Table S2), the AQUA tool evaluation (Table S3), the list of included papers after full text evaluation (Table S4) and the PRISMA checklist (S5) can be found under Supplemental Materials.

## 3. Results

**Figure 1:**
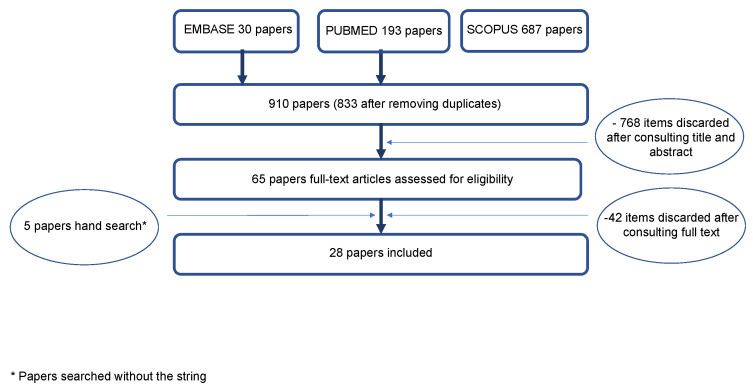
Flowchart of the literature search and selection process. The references were retrieved from the databases of Embase, MEDLINE/PubMed and Scopus.

**Figure 2:**
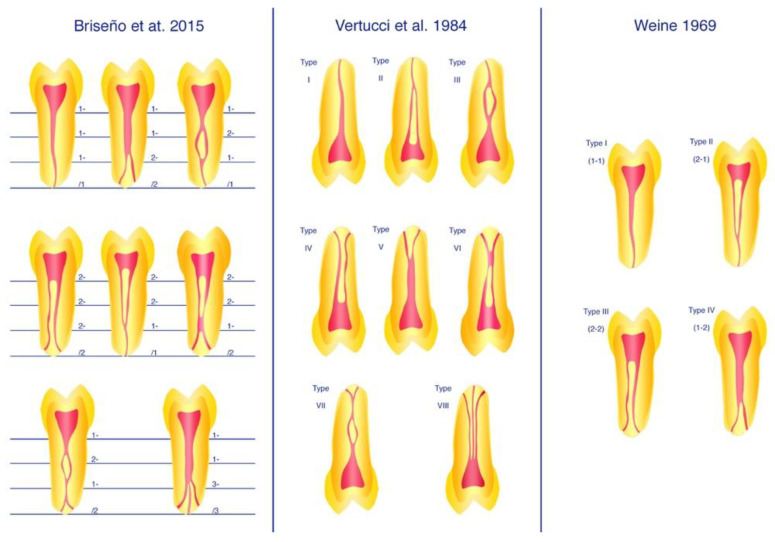
Classification systems proposed by Briseño-Marroquín et al. (2015) [3], Vertucci (1984) [1] and Weine et al. (1969) [2].

The literature search through the three databases (MEDLINE via PubMed, Embase, Scopus) resulted in a total of 910 articles. After all duplicates were removed, the remaining articles (*n* = 833) were examined according to title and abstract, and 768 papers were discarded after consulting the title and abstract. A total of 65 articles were reviewed in full text, and a further 42 papers were excluded after consulting the full text. Through cross-referencing and a hand search of the bibliographies of the full-text articles, another five articles were added to this review. Finally, 28 articles containing randomized controlled trials, cross-sectional studies, comparative studies and evaluation studies from different study populations were included (Figure 1). The classification systems proposed by Briseño-Marroquín et al. (2015) [3], Vertucci (1984) [1] and Weine et al. (1969) [2] are depicted in Figure 2.

Table 1 shows detailed information on the articles: authors, year of publication, sample size, research methods used, number of roots and root canal configurations (RCCs) observed based on the classification systems by Vertucci [1], Weine et al. [2] and Briseño-Marroquín et al. [3].

Only two-thirds of the studies provided information on the number of roots observed. Overall, in accordance with the investigation, single-rooted MaCa were by far the most frequently observed (87.9–100%) [5,7,8,14,16,17,18,19,20,21,23,24,25,27,28,29,30]; two-rooted MaCa rarely occurred (0.0–12%) [7,8,14,16,17,18,19,20,21,23,24,25,27,29,30]. More than two roots were not reported in any of the articles investigated. With a frequency of 35.8% to 96.4%, Briseño-Marroquín’s 1-1-1/1, also known as Vertucci’s I or Weine’s I RCC, is the most common RCC reported [5,6,7,8,9,10,11,12,13,14,15,16,17,18,19,20,21,22,23,24,25,26,27,28,29,30,31,42]. The next most frequent RCCs reported are Briseño-Marroquín’s 2-2-1/1 (Vertucci’s and Weine’s II) [5,7,8,9,10,12,13,14,15,16,17,18,19,20,21,22,23,25,27,28,29,30,31,42] (0.2–22.0%) and Briseño-Marroquín’s 1-2-1/1 (Vertucci’s III) [5,6,8,9,10,12,13,14,16,17,18,19,20,21,22,23,24,26,27,28,29,30,31,42] (0.9–20.0%). Most studies report with a relative low frequency Briseño-Marroquín’s 2-2-2/2 RCC (Vertucci’s IV or Weine’s III) [7,8,9,10,11,12,13,14,15,16,17,20,23,29,30,31,42] (0.0–13.0%) and Briseño-Marroquín’s 1-1-2/2 RCC (Vertucci’s V) [5,8,13,14,17,18,19,20,21,23,24,27,28,29,30,31] (0.2–8.0%). Briseño-Marroquín’s 2-1-2/2 (Vertucci’s VI; 1.0%) [29] and Briseño-Marroquín’s 1-2-1/2 (Vertucci’s VII; 0.1–1.0%) [17,19,21] appear even scarcer while Briseño-Marroquín’s 1-1-3/3 (Vertucci’s VIII) never occurred. This review includes comparative studies that investigated gender differences [9,13,14], different research methods [9], or comparisons between left and right MaCa [26,29,30]. The most commonly used research method reported is the CBCT analysis [6,13,14,16,17,18,19,20,21,22,23,24,25,26,27,28,29,30,31], with the radiographic [15], staining and clearing [5,6,7,8,9,10,42], or cross-sectioning [12] methods less frequently employed. To date, there have been no studies that used the micro-computed tomography technique on root canal configuration in mandibular canines.

## 4. Discussion

The present study was designed and conducted as a systematic review of the root canal configurations of mandibular canines, in order to provide the dentist with knowledge/understanding of the root canal morphology to be expected during clinical treatment.

Various research methods have been used to examine root canal morphologies, such as decalcifying and ink dye [5,6,7,8,9,10,42], radiographic [15], cross-sectional [12], CBCT imaging [6,13,14,16,17,18,19,20,21,22,23,24,25,26,27,28,29,30,31], and micro-CT imaging [34,37]. While the sectioning method requires the destruction of the specimens and, due to the thickness of the slices, an exact reconstruction of the canal anatomy is not possible, radiographic examination is a largely subjective method that is difficult to interpret. Thus, it is not surprising that with current progress in three-dimensional imaging, historical sectioning techniques, as well as conventional two-dimensional radiographs, tend to be being replaced by morphological root canal studies that can be performed using more accurate methods [35].

Several reviewed studies that considered the morphology of the mandibular canines (MaCa) were performed by means of CBCT imaging, examining a relatively large sample size [6,13,14,16,17,18,19,20,21,22,23,24,25,26,27,28,29,30,31]. Although CBCT images do not provide images that are as high-resolution as those of micro-CT, it appears to be a good method to examine root canal configurations [3,43]. Few studies have investigated the MaCa root canal morphology by means of micro-CT [33,34,37,38]. However, those investigating morphological parameters different from the ones in the systematic review investigated other topics than root canal configuration; thus, they did not meet the inclusion criteria and could not be considered in the present study.

The root canal configuration systems proposed by Vertucci [1] and Weine et al. [2] have been extensively used to describe root canal configuration. With computer-assisted imaging techniques, such as micro-CT, it has been possible to depict further root canal configurations; however, these cannot be correctly classified with the stated classification systems by Vertucci [1] and Weine et al. [2].

The present systematic review results show that the 1-1-1/1 RCC is the most common root canal configuration encountered in MaCa [5,6,7,8,9,10,11,12,13,14,15,16,17,18,19,20,21,22,23,24,25,26,27,28,29,30,31,42]. This RCC has been also reported with relatively low frequencies ranging from 35.8% to 62.0% [9,14]. However, most of the articles included in this literature review report a 1-1-1/1 RCC ranging from 76.0% to 96.4% [5,6,7,8,10,11,12,13,15,16,17,18,19,20,21,22,23,24,25,26,27,28,29,30,31,42]. These differences could be explained by the different research evaluation methodologies, unknown gender differences due to anonymous assessment, ethnic origin and the populations investigated. The 1-2-1/2 (14.3%), describing one root canal that splits into two, merges apically and ends with two physiological foramina, was very seldom observed [17,19,21] and only had a 0.1% frequency. The reviewed studies [5,7,8,9,10,12,13,14,15,16,17,18,19,20,21,22,23,25,27,28,29,30,31,42] showed a low number of 2-2-1/1 RCC (0.2–22.0%).

Despite the possible differences and the superiority of the gold standard micro-CT, studies using this method cannot currently be found in the literature for the root canal configuration of the mandibular canine. Further research is needed; the investigation of accessory canals across all root thirds, observed and evaluated mainly with the micro-CT method, could provide additional information and enhance the knowledge of the dentist to increase the success of an endodontic treatment based on additional understanding, improved therapy decisions, and the appropriate selection of instruments and techniques.

## 5. Conclusions

- Mandibular canines are most frequently single-rooted (87.9–100%).
- The most observed RCC is the 1-1-1/1 (Vertucci’s and Weine’s et al. type I), followed by a 2-2-1/1 (Vertucci’s and Weine’s II) and 1-2-1/1 (Vertucci’s III).
- CBCT is widely and, in recent years, most frequently used for *in vivo* research on the root canal morphology of mandibular canines.

## Figures and Tables

**Table 1:** Systematic literature review summary of different comparative and non-comparative morphologic investigations of the root canal configuration (RCC) of mandibular canines. The RCCs are depicted according to the classifications of Weine et al. (We) [2], Vertucci (Ve) [1] and Briseño-Marroquín et al. (Br) [3] (PP: Country three-digit code of population investigated; Met: Research methodology employed; -: no classification given/possible; *: other root canal configurations; CHN*: Chinese subpopulation; Cl: clearing method; Rx: radiographic method; GR: grinding method; SC: staining and clearing (Mic: under microscopic observation); CR: cross-sectional method: T-33 = mandibular left canine; T-43: mandibular right canine; Cr: cross-sectional method; CBCT: cone-beam computed tomography; m-CT: micro CT; F: female; M: male; (l): left; (r): right).

Report	PP	*n*	Met	Root Canal Configuration Frequency (%)									Number of Roots (%)	
	Root Canal Configuration		Ve	I	II	III	IV	V	VI	VII	VIII	*	1	2
			We	I	II	-	III	-	-	-	-	*		
			Br	1-1-1/1	2-2-1/1	1-2-1/1	2-2-2/2	1-1-2/2	2-1-2/2	1-2-1/2	1-1-3/3	*		
Pineda and Kuttler, 1972 [15]	MEX	187	Rx	81.5	13.5	-	5.0	-	-	-	-	-	-	-
Green et al., 1973 [11]	USA	100	GR	87.0	-	-	13	-	-	-	-	-	-	-
Vertucci, 1974 [10]	USA	100	SC	78.0	14.0	2.0	6.0	-	-	-	-	-	-	-
Pécora et al., 1993 [7]	BRA	830	SC	92.2	4.9	-	1.2	-	-	-	-	-	98.3	1.7
Caliskan et al., 1995 [5]	TUR	100	SC	80.4	3.92	13.7	-	2.0	-	-	-	-	100	-
Sert et al., 2004 [42]	TUR	200	SC	76.0	16.0	6.5	1.5	-	-	-	-	-	-	-
Sert and Bayirli, 2004 [9]	TUR	200	M	90.0	9.0	-	-	-	-	-	-	-	-	-
			SC; Mic	-	-	-	-	-	-	-	-	1.0	-	-
			F	62.0	22.0	13.0	3.0	-	-	-	-	-	-	-
Bakianian Vaziri et al., 2008 [12]	IRN	100	CR	88.0	5.0	7.0	-	-	-	-	-	-	-	-
Aminsobhani et al., 2013 [14]	IRN	608	M	36.0 ± 0.3	5.1 ± 0.2	1.4 ± 0.1	6.4 ± 0.2	1.3 ± 0.1	-	-	-	-	-	-
			CBCT	-	-	-	-	-	-	-	-	-	96.3	4.7
			F	35.8 ± 0.1	5.2 ± 0.3	1.4 ± 0.1	6.4 ± 0.1	1.0 ± 0.2	-	-	-	-	-	-
Rahimi et al., 2013 [8]	IRN	149	SC	91.6	6.11	2.29	-	-	-	-	-	-	87.9	12.1
Altunsoy et al., 2014 [13]	TUR	1604	M	91.0	2.6	1.5	0.9	3.5	-	-	-	-	-	-
			CBCT	-	-	-	-	-	-	-	-	-	-	-
			F	94.0	1.6	0.9	1.8	1.8	-	-	-	-	-	-
Han et al., 2014 [20]	CHN*	1291	CBCT	93.7	0.62	3.25	-	0.54	-	-	-	-	98.7	1.3
Somalinga Amardeep et al., 2014 [28]	IND	250	CBCT	79.6	3.2	13.6	-	2.0	-	-	-	1.6	100	-
Zhengyan et al., 2015 [30]	CHN	1452	CBCT/T-33	96.4	0.7	1.7	-	0.4	-	-	-	-	99.2	0.8
		1435	CBCT/T-44	95.2	0.7	2.5	0.3	0.4	-	-	-	-		
da Silva et al., 2016 [31]	BRA	200	CBCT	90.5	1.0	4.0	2.5	2.0	-	-	-	-	-	-
Haghanifar et al., 2017 [19]	IRN	365	CBCT	88.2	3.3	8.1	-	0.3	-	0.1	-	-	99.7	0.3
Martins et al., 2017 [23]	PRT	1200	CBCT	90.2	3.3	2.7	1.4	2.3	-	-	-	0.1	97.2	2.8
Raman et al., 2017 [26]	IND	100	CBCT/T-33	78.0	-	20.0	-	-	-	-	-	-	-	-
		100	CBCT/T-43	84.0	-	14.0	-	-	-	-	-	-	-	-
Soleymani et al., 2017 [27]	IRN	300	CBCT	89.7	3.7	5.7	-	1.0	-	-	-	-	98.7	1.3
Al-Dahman et al., 2019 [16]	SAU	454	CBCT	95.4	2.6	1.8	0.2	-	-	-	-	-	99.8	0.2
Mashyakhy, 2019 [24]	SAU	410	CBCT	90.7	-	6.1	-	3.2	-	-	-	-	97.3	2.7
Naseri et al., 2019 [6]	IRN	30	CBCT	93.9	-	6.1	-	-	-	-	-	-	-	-
			SC	90.9	-	9.1	-	-	-	-	-	-	-	-
Pan et al., 2019 [25]	MYS	411	CBCT	95.1	4.9	-	-	-	-	-	-	-	98.8	1.2
Doumani et al., 2020 [18]	SYR	418	CBCT	95.9	0.73	3.18	-	0.24	-	-	-	-	97.9	2.2
Karobari et al., 2020 [21]	MYS	1702	CBCT	90.7	0.2	8.2	-	0.7	-	0.1	-	0.4	99.7	0.3
Kulkarni et al., 2020 [22]	USA	259	CBCT	85.0	14.0	1.0	-	-	-	-	-	-	-	-
Sroczyk-Jaszczyńska et al., 2020 [29]	POL	100	CBCT/T-33	82.0	4.0	4.0	1.0	8.0	-	-	-	-	92.0	8.0
		104	CBCT/T-43	88.2	-	3.85	-	5.88	0.98	-	-	0.98	96.2	3.9
Candeiro et al., 2021 [17]	BRA	4805	CBCT	89.1	1.58	6.66	0.10	2.41	-	0.13	-	-	97.6	2.4

## Data Availability

The data presented in this study are available in Supplementary Materials (Table S1: List of excluded papers, Table S2: Quality Assessment, Table S3: AQUA Tool Evaluation, Table S4: List of included papers after full text evaluation, S5: PRISMA checklist.)

## Supplementary Materials

The following are available online at https://www.mdpi.com/article/10.3390/ijerph181910197/s1, Table S1: List of excluded papers, Table S2: Quality assessment, Table S3: AQUA tool evaluation, Table S4: List of included papers after full text evaluation, S5: PRISMA checklist.

## References

[1] Vertucci F.J. (1984). Root canal anatomy of the human permanent teeth. Oral Surg. Oral Med. Oral Pathol..

[2] Weine F.S., Healey H.J., Gerstein H., Evanson L. (1969). Canal configuration in the mesiobuccal root of the maxillary first molar and its endodontic significance. Oral Surg. Oral Med. Oral Pathol..

[3] Briseno-Marroquin B., Paque F., Maier K., Willershausen B., Wolf T.G. (2015). Root Canal Morphology and Configuration of 179 Maxillary First Molars by Means of Micro-computed Tomography: An Ex Vivo Study. J. Endod..

[4] Gulabivala K., Aung T.H., Alavi A., Ng Y.L. (2001). Root and canal morphology of Burmese mandibular molars. Int. Endod. J..

[5] Çalişkan M.K., Pehlivan Y., Sepetçioǧlu F., Türkün M., Tuncer S.Ş. (1995). Root canal morphology of human permanent teeth in a Turkish population. J. Endod..

[6] Naseri M., Ahangari Z., Bagheri N., Jabbari S., Gohari A. (2019). Comparative Accuracy of Cone-Beam Computed Tomography and Clearing Technique in Studying Root Canal and Apical Morphology of Mandibular Canines. Iran. Endod. J..

[7] Pécora J.D., Sousa Neto M.D., Saquy P.C. (1993). Internal anatomy, direction and number of roots and size of human mandibular canines. Braz. Dent. J..

[8] Rahimi S., Milani A.S., Shahi S., Sergiz Y., Nezafati S., Lotfi M. (2013). Prevalence of two root canals in human mandibular anterior teeth in an Iranian population. Indian J. Dent. Res..

[9] Sert S., Bayirli G.S. (2004). Evaluation of the root canal configurations of the mandibular and maxillary permanent teeth by gender in the Turkish population. J. Endod..

[10] Vertucci F.J. (1974). Root canal anatomy of the mandibular anterior teeth. J. Am. Dent. Assoc..

[11] Green D. (1973). Double canals in single roots. Oral Surg. Oral Med. Oral Pathol..

[12] Bakianian Vaziri P., Kasraee S., Abdolsamadi H.R., Abdollahzadeh S., Esmaeili F., Nazari S., Vahedi M. (2008). Root Canal Configuration of One-rooted Mandibular Canine in an Iranian Population: An In Vitro Study. J. Dent. Res. Dent. Clin. Dent. Prospect..

[13] Altunsoy M., Ok E., Nur B.G., Aglarci O.S., Gungor E., Colak M. (2014). A cone-beam computed tomography study of the root canal morphology of anterior teeth in a Turkish population. Eur. J. Dent..

[14] Aminsobhani M., Sadegh M., Meraji N., Razmi H., Kharazifard M.J. (2013). Evaluation of the root and canal morphology of mandibular permanent anterior teeth in an Iranian population by cone-beam computed tomography. J. Dent..

[15] Pineda F., Kuttler Y. (1972). Mesiodistal and buccolingual roentgenographic investigation of 7,275 root canals. Oral Surg. Oral Med. Oral Pathol..

[16] Al-Dahman Y., Alqedairi A., Alfawaz H., Alnassar F., Al-Jebaly A. (2019). Cone-beam computed tomographic evaluation of root canal morphology of mandibular canines in a Saudi subpopulation. Saudi Endod. J..

[17] Candeiro G.T.M., Monteiro Dodt Teixeira I.M., Olimpio Barbosa D.A., Vivacqua-Gomes N., Alves F.R.F. (2021). Vertucci’s Root Canal Configuration of 14,413 Mandibular Anterior Teeth in a Brazilian Population: A Prevalence Study Using Cone-beam Computed Tomography. J. Endod..

[18] Doumani M., Habib A., Alhalak A.B., Al-Nahlawi T.F., Al Hussain F., Alanazi S.M. (2020). Root canal morphology of mandibular canines in the Syrian population: A CBCT Assessment. J. Fam. Med. Prim. Care.

[19] Haghanifar S., Moudi E., Bijani A., Ghanbarabadi M.K. (2017). Morphologic assessment of mandibular anterior teeth root canal using CBCT. Acta Med. Acad..

[20] Han T., Ma Y., Yang L., Chen X., Zhang X., Wang Y. (2014). A study of the root canal morphology of mandibular anterior teeth using cone-beam computed tomography in a Chinese subpopulation. J. Endod..

[21] Karobari M.I., Noorani T.Y., Halim M.S., Ahmed H.M.A. (2021). Root and canal morphology of the anterior permanent dentition in Malaysian population using two classification systems: A CBCT clinical study. Aust. Endod. J..

[22] Kulkarni V., Duruel O., Ataman-Duruel E.T., Tozum M.D., Nares S., Tozum T.F. (2020). In-depth morphological evaluation of tooth anatomic lengths with root canal configurations using cone beam computed tomography in North American population. J. Appl. Oral Sci..

[23] Martins J.N.R., Marques D., Mata A., Caramês J. (2017). Root and root canal morphology of the permanent dentition in a Caucasian population: A cone-beam computed tomography study. Int. Endod. J..

[24] Mashyakhy M. (2019). Prevalence of a Second Root and Canal in Mandibular and Maxillary Canines in a Saudi Arabian Population: A Cone-beam Computed Tomography Study. J. Contemp. Dent. Pr..

[25] Pan J.Y.Y., Parolia A., Chuah S.R., Bhatia S., Mutalik S., Pau A. (2019). Root canal morphology of permanent teeth in a Malaysian subpopulation using cone-beam computed tomography. BMC Oral Health.

[26] Raman S., Kumar V.J. (2017). A cone-beam computed tomography study of the prevalence of two or more canals in mandibular anteriors in the Chennai population. J. Adv. Pharm. Educ. Res..

[27] Soleymani A., Namaryan N., Moudi E., Gholinia A. (2017). Root Canal Morphology of Mandibular Canine in an Iranian Population: A CBCT Assessment. Iran. Endod. J..

[28] Somalinga Amardeep N., Raghu S., Natanasabapathy V. (2014). Root canal morphology of permanent maxillary and mandibular canines in Indian population using cone beam computed tomography. Anat. Res. Int..

[29] Sroczyk-Jaszczyńska M., Kołecki J., Lipski M., Puciło M., Wilk G., Falkowski A., Kot K., Nowicka A. (2020). A study of the symmetry of roots and root canal morphology in mandibular anterior teeth using cone-beam computed tomographic imaging in a Polish population. Folia Morphol..

[30] Zhengyan Y., Keke L., Fei W., Yueheng L., Zhi Z. (2016). Cone-beam computed tomography study of the root and canal morphology of mandibular permanent anterior teeth in a Chongqing population. Ther. Clin. Risk Manag..

[31] Da Silva E.J., de Castro R.W., Nejaim Y., Silva A.I., Haiter-Neto F., Silberman A., Cohenca N. (2016). Evaluation of root canal configuration of maxillary and mandibular anterior teeth using cone beam computed tomography: An in-vivo study. Quintessence Int..

[32] Lee K.W., Kim Y., Perinpanayagam H., Lee J.K., Yoo Y.J., Lim S.M., Chang S.W., Ha B.H., Zhu Q., Kum K.Y. (2014). Comparison of alternative image reformatting techniques in micro-computed tomography and tooth clearing for detailed canal morphology. J. Endod..

[33] Marceliano-Alves M.F., de Lima C.O., Augusto C.M., Almeida Barbosa A.F., Vieira Bruno A.M., Rosa A.M., Lopes R.T. (2018). The internal root canal morphology of single-rooted mandibular canines revealed by micro-computed tomography. J. Conserv. Dent..

[34] Mazzi-Chaves J.F., Silva-Sousa Y.T.C., Leoni G.B., Silva-Sousa A.C., Estrela L., Estrela C., Jacobs R., Sousa-Neto M.D. (2020). Micro-computed tomographic assessment of the variability and morphological features of root canal system and their ramifications. J. Appl. Oral Sci..

[35] Plotino G., Grande N.M., Pecci R., Bedini R., Pameijer C.H., Somma F. (2006). Three-dimensional imaging using microcomputed tomography for studying tooth macromorphology. J. Am. Dent. Assoc..

[36] Rhodes J.S., Ford T.R., Lynch J.A., Liepins P.J., Curtis R.V. (1999). Micro-computed tomography: A new tool for experimental endodontology. Int. Endod. J..

[37] Versiani M.A., Pécora J.D., Sousa-Neto M.D. (2013). Microcomputed tomography analysis of the root canal morphology of single-rooted mandibular canines. Int. Endod. J..

[38] Wang M., Ren X., Pan Y. (2019). Micro-computed tomography-based anatomical study of the branch canals in mandibular anterior teeth in a Chinese population. Clin. Oral Investig..

[39] Da Silva Ramos Fernandes L.M.P., Rice D., Ordinola-Zapata R., Alvares Capelozza A.L., Bramante C.M., Jaramillo D., Christensen H. (2014). Detection of various anatomic patterns of root canals in mandibular incisors using digital periapical radiography, 3 cone-beam computed tomographic scanners, and micro-computed tomographic imaging. J. Endod..

[40] Liberati A., Altman D.G., Tetzlaff J., Mulrow C., Gøtzsche P.C., Ioannidis J.P., Clarke M., Devereaux P.J., Kleijnen J., Moher D. (2009). The PRISMA statement for reporting systematic reviews and meta-analyses of studies that evaluate healthcare interventions: Explanation and elaboration. BMJ.

[41] Henry B.M., Tomaszewski K.A., Ramakrishnan P.K., Roy J., Vikse J., Loukas M., Tubbs R.S., Walocha J.A. (2017). Development of the anatomical quality assessment (AQUA) tool for the quality assessment of anatomical studies included in meta-analyses and systematic reviews. Clin. Anat..

[42] Sert S., Aslanalp V., Tanalp J. (2004). Investigation of the root canal configurations of mandibular permanent teeth in the Turkish population. Int. Endod. J..

[43] Wolf T.G., Stiebritz M., Boemke N., Elsayed I., Paqué F., Wierichs R.J., Briseño-Marroquín B. (2020). 3-dimensional Analysis and Literature Review of the Root Canal Morphology and Physiological Foramen Geometry of 125 Mandibular Incisors by Means of Micro-Computed Tomography in a German Population. J. Endod..

