# Supplementary material for: Root Canal Morphology and Configuration of the Mandibular Canine: A Systematic Review
Source: Int J Environ Res Public Health. 2021 Sep 28;18(19):10197. doi: 10.3390/ijerph181910197 (PMC8507995; doi:10.3390/ijerph181910197)
Supplement: Supplementary file 1 [file ijerph-18-10197-s001.zip › S3.AQUA tool evaluation.pdf]

Supplement - AQUA Tool evaluation

Study	Risk of bias				
	**Target and subject attributed**	**Design of the study**	**Methodology description**	**Descriptive anatomy**	**Reporting of outcomes**
Pineda & Kuttler, 1972	Low	Low	Low	Low	Low
Green et al., 1973	Unclear	Low	Unclear	Low	Low
Vertucci, F., 1974	Low	Low	Low	Low	Low
Pécora et al., 1993	Unclear	Low	Unclear	Unclear	Low
Çalişkan et al., 1995	Unclear	Low	Low	Low	Low
Sert et al., 2004	Low	Low	Low	Low	Low
Sert & Bayirli, 2004	Low	Low	Low	Low	Low
Bakianian Vaziri et al., 2008	Unclear	Low	High	High	Unclear
Aminsobhani et al., 2013	Unclear	Low	High	High	Low
Rahimi et al., 2013	Unclear	Low	High	High	High
Altunsoy et al., 2014	Low	Low	Low	Low	Low
Han et al., 2014	Low	Low	Low	Low	Low
Somalinga Amardeep et al., 2014	Unclear	Low	Unclear	Low	Low
Zhengyan et al., 2015	Low	Low	Low	Low	Low
Nogueira Leal da Silva et al., 2016	Low	Low	Unclear	Low	Low
Haghanifar et al., 2017	Low	Low	High	High	Low
Martins et al., 2017	Low	Low	Unclear	Low	Low

Raman et al., 2017	Low	Low	High	High	High
Soleymani et al., 2017	Low	Low	High	High	Low
Al-Dahman et al., 2019	Low	Low	Low	Low	Low
Mashyakhy, M., 2019	Low	Low	Low	Low	Low
Naseri er al., 2019	Unclear	Low	Low	Low	Low
Pan et al., 2019	Low	Low	Unclear	Low	Low
Doumani et al., 2020	Low	Low	High	High	Low
Karobari et al., 2020	Low	Low	Low	High	Unclear
Kulkarni et al., 2020	Low	Low	High	High	Low
Sroczyk-Jaszczyńska et al., 2020	Low	Low	Unclear	Low	Low
Candeiro et al., 2021	Low	Low	Low	Low	Low
