# Supplementary material for: Root Canal Morphology and Configuration of the Mandibular Canine: A Systematic Review
Source: Int J Environ Res Public Health. 2021 Sep 28;18(19):10197. doi: 10.3390/ijerph181910197 (PMC8507995; doi:10.3390/ijerph181910197)
Supplement: Supplementary file 1 [file ijerph-18-10197-s001.zip › S4.List of included papers after full text evaluation.pdf]

Table General characteristics of the included studies mandibular canine

ID	Author	Sources	Type of study	Teeth	Method of study	Country
1	Pineda & Kuttler	Oral Surgery, Oral Medicine, Oral Pathology **1972**, 33(1): 101-110.	In vitro study	Permanent teeth	Rx	Mexico
2	Green et al	Oral Surgery, Oral Medicine, Oral Pathology **1973**, 35(5): 689-696.	In vitro	Permanent teeth	Grinding/examination under X3 magnification	USA
3	Vertucci, et al.	Oral Surgery, Oral Medicine, Oral Pathology **1984**, 58(5): 589-599.	In vitro radiographic	Permanent teeth	Decalcification/dye	USA
4	Pécora et al.	Braz Dent J **1993**, 4(1):53-7.	In vitro	Mandibular Canines	Decalcification/darkended gelatine	Brazil
5	Calişkan et al.	Journal of Endodontics **1995**, 21(4): 200-204.	In vitro study	Permanent teeth	Stereomicroscope, X12 magnification	Turkey
6	Sert et al.	Journal of Endodontics **2004,** 30(7): 494-9.	In vitro	Permanent teeth	Demineralization/ink	Turkey
7	Sert & Bayirli	Journal of Endodontics **2004,** 30(6): 391-398.	In vitro	Permanent teeth	Demineralization/ink	Turkey
8	Bakianian Vaziri et al.	J Dent Res Dent Clin Dent Prospects **2008**, 2(1): 28-32.	In vitro	Mandubular permanet canines	Cross-sections/Stereomicroscope	Iran
9	Aminsobhani et al.	J Dent (Tehran) **2013**, 10(4): 358-66.	Retrospective study	Permanent anterior teeth	CBCT	Iran
10	Rahimi et al.	Indian J Dent Res 2**013,** 24(2): 234-6.	In vitro	Mandibular anterior teeth	Clearing/ Indian ink	Iran
11	Altunsoy et al.	Eur J Dent **2014**, 8(3): 302-306.	Retrospective study	Anterior teeth	CBCT	Turkey
12	Han et al.	Journal of Endodontics **2014**, 40(9): 1309-14.	Retrospective study	Mandibular anterior teeth	CBCT	China (Subpopulation)
13	Somalinga Amardeep et al.	Anat Res Int **2014**, (2014): 7.	In vitro	Maxillary and mandibular canines	CBCT	India
14	Zhengyan et al.	Therapeutics and Clinical Risk Management **2015**,12: 19-25.	Retrospective study	Mandibular permanent anterior teeth	CBCT	China
15	Nogueira Leal da Silva et al.	Quintessence Int **2016**, 47(1): 19-24.	In vivo, retrospective study	Maxillary and mandibular anterior teeth	CBCT	Brazil
16	Haghanifar et al.	Acta Med Acad **2017** 46 (2): 85-93.	Cross-sectional study	Mandibular anterior teeth	CBCT	Iran
17	Martins et al.	International Endodontic Journal **2017**, 50(11): 1013-1026.	Retrospective study	Permanent teeth	CBCT	Portugal
18	Raman et al.	Journal of Advanced Pharmacy Education and Research **2017**, 7(2): 92-95.	Retrospective study	Mandibular anterior teeth	CBCT	India
19	Soleymani et al	Iran Endod J **2017**, 12(1): 78-82.	Retrospective study	Mandibular Canines	CBCT	Iran
20	Al-Dahman et al.	Saudi Endodontic Journal **2019**, 9(2): 113-118.	Retrospective, cross-sectional study	Mandibular Canines	CBCT	Saudi Arabia

21	Mashyakhy, M..	J Contemp Dent Pract **2019, 20(7)**: 773-777.	In vivo, retrospective study	Maxillary and mandibular canines	CBCT	Saudi Arabia
22	Naseri et al.	Iranian Endodontic Journal **2019**, 14(4): 271-277.	In vitro	Mandibular canines	CBCT/ Clearing	Iran
23	Pan et al.	BMC Oral Health **2019**, 19(1): 14.	Retrospective study	Permanent teeth	CBCT	Malaysia
24	Doumani et al.	J Family Med Prim Care **2020**, 9(2): 552-555.	In vivo study	Mandibular canines	CBCT	Syria
25	Karobari et al.	Aust Endod J **2020**	Retrospective study	Anterior permanent teeth	CBCT	Malaysia
26	Kulkarni et al.	J Appl Oral Sci **2020**, 28: e20190103.	Retrospective study	Permanent teeth	CBCT	USA
27	Sroczyk-Jaszczyńska et al.	Quintessence international (Berlin, Folia Morphol (Warsz) **2020**, 79(4): 835-844.	Retrospective study	Mandibular anterior teeth	CBCT	Poland
28	Candeiro et al.	Journal of Endodontics **2020**, article in press.	Prevalence study	Mandibular anterior teeth	CBCT	Brazil
